# Melatonin as a Potential Agent in the Treatment of Sarcopenia

**DOI:** 10.3390/ijms17101771

**Published:** 2016-10-24

**Authors:** Ana Coto-Montes, Jose A. Boga, Dun X. Tan, Russel J. Reiter

**Affiliations:** 1 Department of Morphology and Cellular Biology, Medicine Faculty, University of Oviedo, Julian Claveria, s/n, Oviedo 33006, Spain; 2 Department of Cellular and Structural Biology, UTHSCSA, San Antonio, TX 78229, USA; joseantonio.boga@sespa.es (J.A.B.); tan@uthscsa.edu (D.X.T.); reiter@uthscsa.edu (R.J.R.); 3 Service of Microbiology, Hospital Universitario Central de Asturias, Avenida de Roma, s/n, Oviedo 33011, Spain

**Keywords:** melatonin, sarcopenia, frailty, skeletal muscle, aging

## Abstract

Considering the increased speed at which the world population is aging, sarcopenia could become an epidemic in this century. This condition currently has no means of prevention or treatment. Melatonin is a highly effective and ubiquitously acting antioxidant and free radical scavenger that is normally produced in all organisms. This molecule has been implicated in a huge number of biological processes, from anticonvulsant properties in children to protective effects on the lung in chronic obstructive pulmonary disease. In this review, we summarize the data which suggest that melatonin may be beneficial in attenuating, reducing or preventing each of the symptoms that characterize sarcopenia. The findings are not limited to sarcopenia, but also apply to osteoporosis-related sarcopenia and to age-related neuromuscular junction dysfunction. Since melatonin has a high safety profile and is drastically reduced in advanced age, its potential utility in the treatment of sarcopenic patients and related dysfunctions should be considered.

## 1. Introduction

Worldwide estimates predict 2 billion people will be over 65 years old by 2050 [1]. Thus, an increasingly significant percentage of the population demands remedies and treatments for the deleterious processes that age induces. The scientific community is currently at a loss when it comes to meeting these requirements. Aging is a multifactorial process that provokes slow and persistent functional decline. This gradual physiological deterioration becomes disabling for a high percentage of the elderly population, where it impairs quality of life and increases the demands on primary caregivers and healthcare providers. Of all the degenerative processes, the development of limitations in mobility is one of the most common, leading to a reduced capacity for daily living activities, disability and loss of independence. Slow walking speed, together with unintentional weight loss, self-reported exhaustion, weakness (grip strength) and low physical activity, are the criteria that characterize frailty status. This aging phenotype has been described in detail by Fried et al. [2]. This state of frailty also is characterized by a reduced capacity to respond to demands caused by diminishing functional reserve; this puts the individual in a special risk category when facing minor stressors and is associated with poor outcomes (disability, hospitalization and death) [3,4]. 

In older adults, mobility limitations have been defined as the self-reported inability to walk a mile, climb stairs or perform heavy housework [5]. This impaired mobility is very often associated with a well-established factor of age-related decline in muscle mass designated as sarcopenia [6]. Sarcopenia, however, not only refers to muscle mass deterioration; numerous other factors are involved in the reduction in muscle quality associated with aging. These include derangement of skeletal myocytes, vascular dysfunction, reduced aerobic capacity, fat infiltration and a decline in bone mineral density [6,7]. 

The high number of individuals affected by this syndrome or at least by some sarcopenia-related features has caused sarcopenia to reach epidemic proportions. Moreover, there is no effective cure currently available for this condition and likewise no known treatments, even palliative, are available. The need to develop interventions to prevent or treat sarcopenia has been repeatedly claimed in the literature [8,9,10]. In the current brief review, we summarize previous data suggesting that melatonin mayto limit the development of several of the derangements associated with sarcopenia. Melatonin has a variety of beneficial effects that may slow the development or reduce the severity of the deleterious processes which inevitably lead to sarcopenia in aging population. To date, the evidence for melatonin’s efficacy relative to reducing sarcopenia has not been systematically summarized.

## 2. Sarcopenia Syndrome

Sarcopenia is a term derived from the Greek words *sarx* (flesh) and *penia* (loss) that was introduced by Rosenberg [11] and was used to classically describe the decline in muscle mass among older people [7,12]. Currently, sarcopenia is a well-documented condition associated with the impaired mobility that occurs with aging [13]. There is increasingly evidence, however, that not only the decline in muscle mass is responsible for sarcopenia, but also a failure in muscle strength or power (referred to as dynapenia) is commonly associated with sarcopenia [6,14,15]. Both sarcopenia and dynapenia typically increase with advancing age, but there are individuals in whom there is a discrepancy between changes in muscle mass and muscle strength, mainly related to occupational physical activity in their midlife [6]. Such activity appears to delay sarcopenia development, while dynapenia is a more constant factor that compromises wellbeing at old ages [15]. To take into account this discrepancy, a new term (i.e., muscle quality) is being increasingly used, referring to the force generating capacity per unit cross-sectional area [6,16,17]. Muscle quality is negatively affected by several processes.

Sarcopenia and energetic imbalance are characteristics of the physiological framework that explain frailty and its consequences [18]. Walston and Fried suggest that there is some feedback between these components, the so-called frailty cycle. This cycle stems from the physiological changes associated with aging, which results in an imbalance between anabolism and catabolism. This state embraces multiple systems and cellular pathways implicated in age-dependent muscle degeneration (reviewed by [7,14]). Thus, sarcopenic muscle exhibits several cellular dysfunctions which result from oxidative stress, mainly due to mitochondrial dysfunction and a reduction of radical scavenging capability. Also included is a reduction in cellular turnover with a significant decrease in the number of satellite cells, alterations in proteolytic activities including those of the proteasome, autophagic dysregulation and even changes in apoptosis. These cellular derangements are associated or are even part of the more general perturbations also involved in sarcopenia development. These include a decrease in sex hormones [19] and an elevated pro-inflammatory state [20]. Eventually, sarcopenia is related to adipocyte infiltration with increases in both intra- and inter-muscular adipose tissue which significantly contributes to the decline in muscle quality [21]. Additional contributing factors include osteoporosis due to close relationship between muscle and bone, which are a single functional unit [7] and a decline in neurophysiological activity. This relates to the fact that age-related changes in the neuromuscular junction (NMJ) play a key role in musculoskeletal impairment, preceding or following the decline in muscle mass [22].

Collectively, the described alterations are embodied in the term sarcopenia and all are well-established risk factors for the major negative health-related conditions and events that characterize aging, including frailty, disability, institutionalization and mortality [23]. The development of preventive and therapeutic strategies against sarcopenia is considered an urgent need by health professionals. Based on what is known about the actions of melatonin, we propose that this molecule may have the potential to counteract sarcopenic damage or, moreover, may prevent some of the alterations associated with muscle quality loss. Additionally, we cited the published literature that shows the efficacious and beneficial effects of melatonin against the features which constitute the multi-pathology called sarcopenia.

## 3. Why Melatonin?

Melatonin, also known as *N*-acetyl-5-methoxytryptamine, is a derivative of tryptophan, an essential amino acid [24]. It is produced by the pineal gland in a circadian manner with maximal production during the night. It is involved in synchronization of circadian rhythms in physiological functions including sleep timing, blood pressure, seasonal reproduction and many others [25,26,27,28,29]. There is also evidence that all other cells produce melatonin [30,31], continually throughout the day, mainly as an antioxidant and free radical scavenger [32,33,34,35]. Melatonin is present in all biological fluids including cerebrospinal fluid, saliva, bile, synovial fluid, amniotic fluid, and breast milk [36,37]; and perhaps in mitochondria and chloroplasts where it may have the capacity to synthesize and metabolize melatonin itself [31,38]. This molecule has important protective capabilities, mainly based on its high potency as a free radical scavenger, low toxicity and solubility in both aqueous and organic media [30,39]. 

Pineal production and plasma melatonin levels progressively drop during aging [40,41,42] to the extent that in advanced age its levels are almost null. The loss of melatonin during aging may have great importance in the general deterioration that the elderly experience. Several investigations have reported a general improvement in life quality due to melatonin supplementation in older adults [43,44,45]. Moreover, numerous articles relate the age-associated decline in melatonin levels with the development of several diseases [46,47,48].

Melatonin is undoubtedly more than a zeitgeber and an antioxidant molecule since it seems to be essential at the cellular level as a physiological regulator of homeostasis. Its therapeutic applications are numerous, from pediatric [49,50,51] to geriatric diseases [52,53,54]; this includes cancer [55,56], sleep disturbances [57,58] and neurodegenerative diseases [59,60]. 

Several clinical trials with melatonin supplementation as a treatment have been successfully performed [61]. These melatonin treatments have often had positive outcomes in different pathologies: reducing cardiac morbidity [62], controlling adverse effects of chemotherapy [63] and alleviating bipolar disorders [64] among others. Also, melatonin has been used as a treatment with significant success in Duchenne muscular dystrophy [65] where it reduced the muscle degenerative process. Based on previous knowledge about the role of melatonin and sarcopenia (as summarized below), it is likely that melatonin may be effective in treating this condition.

## 4. Cellular Impact of Sarcopenia

Sarcopenia is a highly burdensome geriatric syndrome. The heterogeneity of its clinical correlates and the complexity of its pathogenesis make the development of effective preventive and therapeutic measures difficult. In this section we describe the numerous changes that occur at the cellular level in sarcopenic muscle [66].

### 4.1. Increase in Oxidative Stress and Mitochondrial Alterations

Aging is characterized by an increase in oxidative stress which is exacerbated during sarcopenia development. The relationship of oxidative stress to sarcopenia has been experimentally defined [67,68]. Considering this, theoretically at least, the addition of an antioxidant should produce beneficial effects in this condition. However, not all reactive species are harmful. Certainly, it is well-established that some reactive oxygen species (ROS), reactive nitrogen species (RNS), and a basal level of oxidative stress are essential for cell survival [69]. Oxidant generation, within a hormetic range, is essential for intracellular signaling [70] and optimal force production [71]. Thus, very highly efficient antioxidants may paradoxically be harmful unless their effects on the redox balance are closely titrated [72]. However, melatonin seems not to be a typical radical scavenger and many publications show that melatonin also regulates cellular homeostasis [37] and could even promote the generation of free radicals when necessary [34]. For example, we have shown that when high oxidative stress is necessary for adequate organ development, daily melatonin injections initially induce a reduction of oxidative stress but, subsequently, when the melatonin injections are continued, free radical generation is restored [73]. The collective findings indicate that melatonin is able to reduce free radical concentrations but maintain them inside homeostatic limits and, moreover, melatonin’s action as a free radical scavenger and as antioxidant are context specific as described by Proietti and colleagues [74].

The rise in oxidative stress in sarcopenia is mainly a result of mitochondrial dysfunction. Any derangements in skeletal myocyte mitochondrial function are recognized as major factors that contribute to age-dependent muscle degeneration [67]. In this regard, it is noteworthy that slow walking speed has been adopted as a criterion for defining sarcopenia [75]; this is likely due to a mitochondrial bioenergetic decline during muscle aging [76]. Melatonin and its metabolites are powerful antioxidants protecting the electron transport chain and mitochondrial DNA from oxidative damage more efficiently than other conventional antioxidants [77]. This protection of the respiratory chain allows melatonin to increase ATP production in mitochondria [78].

### 4.2. Cellular Vacuolization: Alterations in Autophagy

The process of vacuolization is currently poorly understood. According to Henics and Wheatley [79], vacuolization is simply the state of being with vacuoles; this implies a continual process of becoming progressively more vacuolated. This occurs in most cell types spontaneously or via a wide range of inductive stimuli. Vacuoles can be formed from several organelle types of the endosomal/lysosomal compartment and is generally considered a degenerative process. The involvement of autophagosomes in vacuole formation is widely accepted [80]. Also, some agents impair autophagy, inducing blockage, which results in vacuole accumulation [81]. Strongly supporting this hypothesis, several articles show functional defects in autophagy as a characteristic of sarcopenic muscle [7,82]; this has been occasionally accompanied by perinuclear accumulation of autophagic vacuoles [83].

Melatonin, in its role as a homeostasis stabilizer, has been shown to induce [84] or reduce [85] autophagy. In relation to muscle, melatonin is highly versatile molecule and either induces autophagy [86] or inhibits it [87], depending on pathological processes involved, since oxidative stress has a close relationship with autophagy. For example, melatonin induces autophagy in myoblast cells collaborating in myogenic differentiation (MyoD) degradation [88] but it inhibits autophagy in muscles from carbon tetrachloride-treated mice by reducing oxidative stress-induced damage [89].

### 4.3. Protein Degradation Deterioration

Sarcopenia is a syndrome where the cell’s catabolic machinery has collapsed or has become misregulated [90]. The accumulation of lipofuscin granules in an increasing number of lysosomes of sarcopenic muscles is an example of impaired lysosomal degradative capacity [91]. In this process, only a small amount of lysosomal enzymes remains available for degradative pathways [67]; this significantly contributes to the reduction in the degenerative capacity of these organelles. On the other hand, higher levels of myostatin, a transforming growth factor-β (TGF-β) family member, induce muscle wasting by activating proteasomal-mediated catabolism of intracellular proteins [92]. In addition, defects in protein synthesis has been detected in muscles of sarcopenic patients [93] 

Melatonin reduces endoplasmic reticulum stress in skeletal muscle by increasing the expression of several proteins as well as mRNA levels [89]; this improves protein synthesis. Likewise, melatonin is an important regulator of proteasome [94] and lysosomal mechanisms [88], thereby enhancing cell quality.

### 4.4. Decrease in Satellite Cells and Increase in Apoptosis

Satellite cells in skeletal muscle are quiescent mono-nucleated myogenic cells, located between the sarcolemma and basement membrane of terminally-differentiated muscle fibres [95]. The life-long maintenance of muscle tissue involves satellite cells, since under homeostatic conditions satellite cells are activated by stimuli such as physical trauma or growth signals [96]. Sarcopenia increases the susceptibility to muscle injury [97] and the reduced muscle mass contributes to falls [98]; in these situations satellite cell activation would be essential for improving regeneration of these old muscles. However, satellite cells are drastically reduced in sarcopenia increasing the negative consequences of sarcopenic muscle [99] and/or its funcionality [100]. Unfortunately, these changes are sarcopenic characteristics [7]. 

Melatonin also increases satellite cells following muscle injury in rats [101] by reducing the apoptotic processes via modulation of signaling pathways which causes significant muscle regeneration in these animals. Antiapoptotic actions of melatonin have been described in many tissues and in a variety of normal cell types [102,103]. However, melatonin’s role in apoptosis can differ among normal and cancer cells, since several publications have shown melatonin’s capability to destroy cancer cells by triggering apoptosis [104,105,106]. In contrast, in normal skeletal muscle, some authors have described in detail how melatonin prevents apoptosis and limits the oxidative stress that causes mitochondria permeability transition and subsequent death [107]. Melatonin, for example, attenuates apoptotic processes during ischemia/reperfusion in skeletal muscle [108]. Considering these findings, melatonin has been proven to significantly reduce or, even, counteract several pathophysiological processes specifically associated with sarcopenia [7].

### 4.5. Chronic Low Inflammation

There are other processes, some of them being a result of the changes described above, which are common to different pathologies and are part of the sarcopenic complex. Melatonin may also counteract or reduce those pathologies. One example is the systemic subacute inflammation which is a predominant characteristic of the aging process [109]. This low grade inflammation has been implicated in the development of a number of chronic diseases [110] and is associated with sarcopenia development as well [67,111]. The damaging agents in this process are notably interleukin 6 (IL-6), C-reactive protein (CRP) and tumor necrosis factor α (TNF-α) [112,113]. Recent evidence has documented a role for melatonin in reducing inflammation in muscle cells, acting specifically against these cytokines in rats [114] and also in humans [115]. The anti-inflammatory actions of melatonin are well-documented in numerous organs [116].

### 4.6. Endocrine Signaling

Studies on the nature and magnitude of age-related perturbations in circulating hormones as well as the responsiveness of target tissues are major features of sarcopenia research [82]. A number of hormone levels change during sarcopenia, including myokines and adipokines, due to the crosstalk between muscle and adipose tissue [117,118]. Also, alterations in the renin–angiotensin system promote muscular inflammation, mitochondrial dysfunction, and apoptosis [119]; insulin resistance leads to perturbed metabolism and misrouted signaling [120]. Also, reductions in testosterone and dehydroepiandrosterone contribute to muscle loss or weakness [121], while growth hormone (GH) and insulin-like growth factor 1 (IGF-1) decrease, which is deleterious to the physical function of skeletal muscle with age [122]. 

Hormonal supplementation in the older adults has been used to restore endocrine signaling. This procedure is controversial and disappointing results in sarcopenic individuals have been obtained [67]. As a result, great disparities between recommendations from scientific societies related to aging and elderly patients in general have been mentioned [121]. Consequently, hormonal supplementation seems not to be a desirable option. As an example, special attention should be paid regarding GH where long-time supplementation as an anti-aging strategy has caused a number of severe side effects associated with this treatment, and the Growth Hormone Research Society has warned against the use of GH or its secretagogues [123]. 

With regard to supplementation with melatonin, firstly, no significant adverse effects have been reported with its use at any concentration or at any treatment time. Also, melatonin, as an effective testosterone substitute, has been shown to prevent muscular atrophy in rats induced by castration through the IGF-1 axis [124]. Moreover, melatonin reduces adipogenesis in obese mice [85], collaborates in insulin resistance attenuation in *Caenorhabditis elegans* [125] and has a regulatory role in autocrine and paracrine responses in muscle and adipose tissue [126]. Additionally, melatonin has been shown to be more effective than GH in recovering physiological functions in smooth muscle from old rats [127].

### 4.7. Vascular Aging

Aging of the vascular system significantly hinders the uptake of oxygen and nutrients by muscle cells; this is closely related to sarcopenia development. Thus, aged skeletal muscle shows reduced blood flow capacity [128] together with extensive damage to endothelium-dependent vasodilation. Both processes promote mitochondrial destruction in muscle cells due to a reduction in microvascular oxygenation [129]; this in turn, induces ATP failure, increases ROS generation that also affects blood vessel integrity. Thus, a vicious cycle involving oxidant production and vascular and muscular damage ensues [67]. 

In contrast, a long-term treatment with melatonin has vasculoprotective properties [130]; for example, it restores vascular dysfunction in a model of accelerated aging (i.e., the senescence accelerated mouse-prone 8 (SAMP8)). Moreover, melatonin improves endothelial damage and causes important improvements in vessel cytoarchitecture in aged animals [131]. Finally, benefits in delaying age-related cellular damage in the cardiovascular system have been observed in aged rats supplemented with caffeic acid phenethyl ester and melatonin [132].

Age-related damage of skeletal muscle cannot be studied as an isolated entity because to its close relation with bone and the involvement of neuromuscular junctions. Unrepaired damage to one of these two systems renders treatments for improving sarcopenia useless. It is essential that melatonin’s capability of restoring the integrity of the musculoskeletal system and neuromuscular junctions also be considered in any attempts to reduce sarcopenia.

## 5. Sarcopenia and Osteoporosis

As mentioned above, bone and muscle are closely interrelated. Thus, when aging affects one of these two tissues, the functionality of the other is likewise compromised [66]. Thus, as muscle quality deteriorates during aging, also bone becomes weakened when it develops osteoporosis.

Osteoporosis literally means “porous bone”. It is a consequence of a reduction in bone mineral density which significantly increases fracture risk, which is the most serious complication of osteoporosis [133]. Muscle force has an important influence on essential bone properties such as mass, size, shape, and, even, architecture [134]. Thus, in elderly sarcopenic patients when the muscle strain falls below a given threshold, bone remodeling activates a so-called disuse mode, which results in less bone formation and greater bone resorption [66]. The reliance of muscle health on bone and vice versa is so interrelated that several researchers consider it one syndrome, with terms including sarco-osteopenia, sarco-osteoporosis, or dysmobility syndrome to distinguish disorders which are prone to a high risk of fractures [66,135,136].

Oxidative stress and autophagic alterations have been implicated in the development of osteoporosis [137], which could account for the beneficial effects of melatonin in this disease [138]. A recent published clinical trial has provided evidence related to the ability of melatonin to improve bone mineral density in humans [139], thereby protecting them against fractures. The ability of melatonin to protect against osteroporosis would also provide benefits in terms of limiting sarcopenia, since elevated bone strength is usually associated with greater muscular tone.

## 6. Neuromuscular Junction

The NMJ is the site at which efferent neurons communicate with muscle fibers. They function in the transmission of signals from the motor neuron to the skeletal muscle fibers to ensure precise control of skeletal muscle contraction and therefore voluntary movement. When the function of the motor neuron terminal is lost, the muscular fiber innervated by this neuron loses its contact to the nervous system and becomes incapable of generating volitional muscle contractions [22]. Although aging is usually associated with a reduction in NMJ function, the mechanisms involved are not well understood. Some lines of evidence point to the changes being causally related to the decline in muscle mass and function as observed in sarcopenia; however, which occurs first, sarcopenia or a reduction in the function of the NMJ, remains unknown [22]. 

Once again, oxidative stress seems to be implicated in NMJ impairment together with mitochondrial dysfunction and inflammation being prominent features [140]. Thus, melatonin, due to its potent antioxidant activities could be a key player in resolving or preventing this deregulation. In fact, published reports using different animals show that melatonin reverses age-related neuromuscular transmission dysfunction [141] and improves, at the same time, muscle physiology [142].

While still limited, the scientific evidence is consistent in terms of suggesting that melatonin significantly improves aged muscle as well as other cellular alterations characteristic of sarcopenia. Melatonin’s action also applies to the pathophysiological processes associated to sarcopenia including muscle dysfunction that is closely interlinked to sarcopenia. Finally, it is necessary to remember that melatonin levels are gradually lost throughout life [41], being almost undetectable in the elderly; this could easily facilitate sarcopenia development. In light of these findings, it is reasonable to assume that maintaining normal endogenous levels of melatonin or administering it as an exogenous supplement may alleviate age-related muscular decline and the development of sarcopenia.

## 7. Conclusions

**Figure 1:**
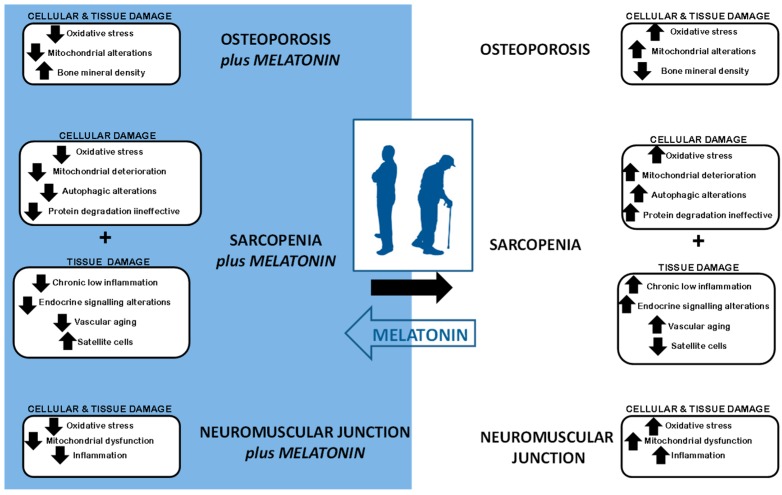
Schematic overview of the potential beneficial effects of melatonin in osteoporosis, sarcopenia and disruption of the neuromuscular junction.

Sarcopenia is a highly burdensome geriatric syndrome. It is commonly associated with osteroporosis and neuromuscular dysfunction. Currently, no effective treatment for this degenerative process has been identified. Melatonin has a high safety profile and no serious toxicity related to melatonin usage has been reported. Here, we summarized the scientific evidence that melatonin prevents and counteracts mitochondrial impairments, reduces oxidative stress and autophagic alterations in muscle cells, increases the number of satellite cells and limits sarcopenic changes in skeletal muscle. Likewise, melatonin lowers chronic low inflammation levels and reduces vascular aging, all of which are usually present in sarcopenic muscle. Similarly, melatonin improves the endocrine signaling which deteriorates in aged individuals. As a consequence, melatonin may be useful to prevent or treat sarcopenia-associated diseases including osteoporosis and neuromuscular dysfunction (Figure 1). Collectively, the published data suggest that melatonin may be a useful aid in slowing age-related muscle deterioration (i.e., sarcopenia as well as its associated conditions). If so, stronger muscles could translate into fewer falls and bone fractures in the older population, which are factors that normally seriously compromise aged individuals’ health.
